# Asprin‐loaded strontium‐containing α‐calcium sulphate hemihydrate/nano‐hydroxyapatite composite promotes regeneration of critical bone defects

**DOI:** 10.1111/jcmm.15918

**Published:** 2020-11-07

**Authors:** Yi Jiang, Hanjun Qin, Haoyang Wan, Jun Yang, Qi Yu, Mo Jiang, Bin Yu

**Affiliations:** ^1^ Department of Orthopaedics Nanfang Hospital, Southern Medical University Guangzhou, Guangdong China; ^2^ Key Laboratory of Bone and Cartilage Regeneration Medicine Southern Medical University Guangzhou, Guangdong China; ^3^ Department of Orthopaedics The First Affiliated Hospital of Nanchang University Nanchang, Jiangxi China; ^4^ Department of Orthopaedics Affiliated Hospital of Jiangxi University of Traditional Chinese Medicine Yingtan, Jiangxi China

**Keywords:** aspirin, bone graft substitute, calcium sulphate hemihydrate, nano‐hydroxyapatite, osteoconductive, osteoinductive, strontium

## Abstract

Our laboratory originally synthesized strontium(Sr)‐containing α‐calcium sulphate hemihydrate/nano‐hydroxyapatite composite (Sr‐α‐CSH/n‐HA) and demonstrated its ability to repair critical bone defects. This study attempted to incorporate aspirin into it to produce a better bone graft material for critical bone defects. After 5% Sr‐α‐CSH was prepared by coprecipitation and hydrothermal methods, it was mixed with aspirin solution of different concentrations (50 μg/ml, 200 μg/ml, 800 μg/ml and 3200 μg/ml) at a fixed liquid‐solid ratio (0.54 v/w) to obtain aspirin‐loaded Sr‐α‐CSH/n‐HA composite. In vitro experiments were performed on the composite extracts. The tibial defects (3 mm*5 mm) in SD rat model were filled with the composite for 4 weeks and 12 weeks to evaluate its osteogenic capacity in vivo. Our results showed its capability of proliferation, migration and osteogenesis of BMSCs in vitro got improved. In vivo treatment with 800 μg/ml aspirin–loaded Sr‐α‐CSH/n‐HA composite led to significantly more new bone formation in the defects compared with Sr‐α‐CSH/n‐HA composite and significantly promoted the expression of osteogenic‐related genes and inhibited osteoclast activity. In general, our research suggests that aspirin‐loaded Sr‐α‐CSH/n‐HA composite may have a greater capacity of repairing tibial defects in SD rats than simple Sr‐α‐CSH/n‐HA composite.

## 1 INTRODUCTION

Tumour resection, malformation, sports injury and infection can damage normal musculoskeletal system, leading to bone defects.^1^, ^2^, ^3^, ^4^ More than 1.5 million patients undergo bone graft surgery every year in the world.^5^ Despite significant advances in bone graft techniques in recent years, critical bone defects still pose enormous challenges to their morphological and functional reconstruction which is a very complex process. For decades, with joint efforts by researchers and clinicians, new methods of bone tissue engineering and new bone graft techniques have been introduced to solve this problem.

There are a wide variety of bone graft substitutes (BGS) used in bone tissue engineering, such as autologous bone, allogeneic bone, xenogeneic bone and various biosynthetic bone. As the gold standard treatment for bone defects, autogenous bone grafting has demonstrated advantages in bone healing without immune rejection. However, limited availability of proper materials and likely complications at donor site like bleeding and infection have forced researchers to discover alternatives. As a substitute for classic grafts, biosynthetic materials can have an unrestricted supply and avoid autogenous bone‐related drawbacks.^6^ Novel engineered bone strives to meet various regenerative requirements of bone tissue simultaneously. Previous studies have demonstrated that defects of single‐component material are inevitable, indicating a necessity to combine two or more materials to achieve better outcome.^7^ Attempts have been made to develop BGSs with antibacterial properties to control infection and increase their rate of successful implantation. It has been reported that antibiotics can be released for 20 days or more continuously in animal models using biodegradable polymer scaffolds containing gentamicin and vancomycin and that local delivery also prevents systemic side effects.^8^ Moreover, synthetic materials can be loaded with various osteoinductive factors such as bone morphogenetic protein (BMP), insulin‐like growth factor (IGF) and transforming growth factor‐β (TGF‐β) to enhance their osteoinductive ability for better bone formation and bone remodelling.^9^, ^10^, ^11^, ^12^

α‐calcium sulphate hemihydrate (α‐CSH), a class of highly cementitious bone substitute widely used in clinic, has shown to possess superior biocompatibility, biodegradability and osteoconductivity. Recent studies have combined α‐CSH with other bioactive inorganic materials to improve its osteogenicity and physicochemical properties.^13^, ^14^, ^15^ Our laboratory previously synthesized strontium(Sr)‐containing α‐hemihydrate calcium sulphate (Sr‐α‐CSH) which had a benefit of osteoinductivity in addition to the bone conductivity and biocompatibility of calcium sulphate.^16^ However, its rate of degradation was too fast. To solve this problem, our laboratory conducted a further research where Sr‐α‐CSH was combined with nano‐hydroxyapatite (n‐HA). The new Sr‐α‐CSH/n‐HA composite showed good biocompatibility, osteoinductivity and improved degradation rate, but its bone support was weak because its compressive strength decreased significantly with increased concentration of strontium.^17^ To obtain a better BGS, we next tried to load aspirin into Sr‐α‐CSH/n‐HA to decrease its concentration of Sr to improve its performance in dealing with critical bone defects.

Aspirin, the most active component of the non‐steroidal anti‐inflammatory medication, is a very popular antipyretic, anti‐inflammatory and analgesic drug. Its other roles in treating diseases have been found. It has a certain impact on bone metabolism and bone health, promoting osteogenic differentiation of bone marrow mesenchymal stem cells (BMSCs), inhibiting adipogenic differentiation of BMSCs, activating osteoblasts and inhibiting osteoclasts.^18^, ^19^, ^20^, ^21^, ^22^ It has been found to promote bone formation by inhibiting the expression of inflammatory factors such as IFN‐γ and TNF‐α.^23^, ^24^, ^25^ It may also improve bone marrow microenvironment and enhance immune regulation of BMSCs. We thus hypothesized that incorporation of aspirin into Sr‐α‐CSH/n‐HA composite might produce a better BGS for critical bone defects.

The purpose of this study is to combine Sr containing α‐CSH/nano‐hydroxyapatite (n‐HA) composite with different concentrations of aspirin, and to test the structure, physicochemical properties, biocompatibility and ability to stimulate the proliferation, migration and differentiation of BMSCs in vivo and in vitro. In addition, the material was implanted into an established tibia bone defect model in SD rats for analysis. Our present study demonstrated that aspirin‐loaded strontium‐containing α‐calcium sulphate hemihydrate/nano‐hydroxyapatite composite has a potential of a proper substitute for classic grafts in treatment of critical bone defects.

## 2 MATERIALS AND METHODS

### 2.1 Preparation of aspirin‐loaded Sr‐α‐CSH/n‐HA composites

Firstly, Sr‐α‐CSH containing 5 mol% Sr (appointed as 5% Sr‐α‐CSH) was prepared in the same way as before (using coprecipitation and hydrothermal methods).^16^ 5% Sr‐α‐CSH and n‐HA (aladdin) was then mixed at a fixed mass ratio (Sr‐α‐CSH: n‐HA = 6:4) to get the Sr‐α‐CSH/n‐HA composite. Secondly, aspirin solution of different concentrations (50 μg/ml, 200 μg/ml, 800 μg/ml and 3200 μg/ml) was prepared with distilled water. Thirdly, aspirin solution of different concentrations was added into the mixture at a fixed liquid‐solid ratio (0.54 v/w). Cylindrical Sr‐α‐CSH/n‐HA composite samples were then obtained by filling, drying and moulding (Composite specifications: φ5 mm × 10 mm, φ3 mm × 5 mm, diameter × height).

### 2.2 Characterization and physicochemical properties of aspirin‐loaded SR‐α‐CSH/N‐HA composite

The crystal structure of the composite material was analysed by XRD (Bruker, D8, Germany). Aspirin‐loaded Sr‐α‐CSH/n‐HA composite was ground for testing. Different materials had their specific crystal structure and unique parameters such as lattice type and interplanar spacing. The characteristics of crystallographic reflection followed Bragg's law (Parameters: current 40 mA, voltage 40 kV, continue scan mode, scan speed 0.75°/s, scan mode θ/2θ, 2θ range 10‐80°). The infrared absorption spectra of different materials varied. FTIR (EQUINOX 55, Bruker, Germany) was used to analyse the main functional groups. According to the requirement for analysis of inorganic samples, the composite was detected after being ground into powder form (scan range: 4000‐5000 cm^−1^; scan frequency: 32 times/second). Data analysis and charting used Origin 8.0 software. A tensile test machine (MTS858, MTS Systems Corporation, USA) was used to analyse the CST of cylindrical composite (material specifications: 5 mm × 10 mm, diameter × height). The compression speed was 1 mm/min and the compressive strength equalled maximum compressive force divided by body surface area (unit: MPa). Five samples from each experimental group were used to calculate the mean and standard deviation of the results. SEM (Phenom XL G2 Netherlands) was used to observe the microstructure of the composite. The experimental sample used to be tested before SEM was pre‐treated according to the following procedures (Vacuum drying and gold spraying).

### 2.3 Preparation of aspirin‐loaded Sr‐α‐CSH/n‐HA composite extracts

The aspirin‐loaded Sr‐α‐CSH/n‐HA composite extracts were prepared according to international standards.^16^ In brief, the composite (0.2 g/ml) was soaked in alpha minimum essential medium (α‐MEM, Invitrogen) cell culture medium at 37°C for 24 hours. After centrifugation, the supernatant was filtered using a 0.22 µm microporous membrane filter to get the composite extract. In in vitro experiments, BMSCs were treated with various extracts of aspirin‐loaded Sr‐α‐CSH/n‐HA composite. Aspirin‐loaded Sr‐α‐CSH/n‐HA composite extracts of different concentrations (0, 50, 200, 800 and 3200 μg/ml) were prepared and then incubated in supplements with 15% FBS, 2 mM L‐glutamine (Invitrogen), 55 mM 2‐mercaptoethanol (Invitrogen), 100 U/mL penicillin and 100 mg/mL streptomycin (Invitrogen) at 37℃ and 5% CO2 in a humidified environment.

### 2.4 Isolation and culture of BMSCs

Isolation of BMSCs from the SD rats was done following the established protocol.^24^ In brief, after the femurs and tibias were removed, the bone marrow cells were flushed out from the bone cavity of femurs and tibias with growth medium for BMSCs carefully. After all the bone marrow cells passed through a 70‐μm cell strainer (BD Bioscience), single‐cell suspension of all nuclear cells was obtained. Thirty to fifty million cells were seeded onto 10cm culture dishes (Corning) for initial incubation for 48 hours with a‐MEM supplemented with 15% FBS, 2 mM L‐glutamine (Invitrogen), 55 mM 2‐mercaptoethanol (Invitrogen), 100 U/mL penicillin and 100 mg/mL streptomycin (Invitrogen) at 37℃ and 5% CO2 in a humidified environment. Cells were passaged once they became 70% to 80% confluent. BMSCs at passage 3 were used in our study.

### 2.5 Cell proliferation assay

The effects of composite extracts on BMSCs proliferation were assessed using the CCK‐8 (Dojindo) assay. In vitro expanded BMSCs were seeded at passage 3 (3.0 × 10^4^ cells/well) in triplicate using a 96‐well flat‐bottom plate (Costar, Cambridge, MA, USA) and maintained in 100 μl medium with aspirin‐loaded Sr‐α‐CSH/n‐HA composite extracts (0 μg/ml, 50 μg/ml, 200 μg/ml, 800 μg/ml and 3200 μg/ml) for 2, 4 and 6 days. At each time point, the cells were treated with 10 mg/ml of CCK‐8 reagent (Sigma‐Aldrich, St. Louis, MO, USA) and incubated at 37°C for 2 hours. Then the absorbance in each well was measured at a wavelength of 450 nm using an automatic enzyme‐linked immunosorbent assay (ELISA) reader (ELx800; BioTek Instruments Inc, Winooski, VT, USA).

### 2.6 Osteogenic differentiation evaluation

Osteogenic differentiation evaluation was performed as previously reported.^26^ Osteogenesis mineralization of BMSCs by composite extract was detected using Alizarin red staining. The gene expression of RUNX2, OCN and BSP was assayed by real‐time PCR (RT‐PCR). All mRNA quantification data represent the mean ± standard error of the mean of triplicate experiments.

### 2.7 In vitro scratch test

BMSCs (1.5 × 10^5^ cells/well) were plated in six‐well plates for 24 hours and wounded by scratching with a 10 μL pipette tip. Cellular debris was removed by washing with PBS and then incubated in a‐MEM (Invitrogen) supplemented with 5% FBS, 2 mM L‐glutamine (Invitrogen), 55 mM 2‐mercaptoethanol (Invitrogen), 100 U/mL penicillin and 100 mg/mL streptomycin (Invitrogen) at 37℃ and 5% CO2 in a humidified environment. The width of the scratch was determined microscopically immediately after creation and 6, 24, 48 hours later using a phase‐contrast microscope (Olympus, Tokyo, Japan). The wounded areas were quantified as wound width by Photoshop (PS).

### 2.8 Transwell assay

The migration assay was tested using transwell plates (Corning Costar, USA) that were 6.5 mm in diameter with 8 μm pore filters. The upper chambers were loaded with 1 × 10^5^ BMSCs in 200 μl of DMEM containing 0.1% BSA, and the lower ones with 500 μl of DMEM containing 10% FBS with different concentrations of composite extracts added. Following incubation for 15 hours, BMSCs in the upper chamber were removed to fix their membranes with 4% paraformaldehyde for 20 minutes. The cells that migrated to the lower side of the filter were stained with 0.1% crystal violet for 10 minutes before observation under a light microscope.

### 2.9 Generation and transplantation of aspirin‐loaded Sr‐α‐CSH/n‐HA composite implanted in critical bone defects of the tibia

Male Sprague Dawley (SD) rats, 8‐week‐old, were purchased from Laboratory Animal Center of Southern Medical University, Guangzhou..The animal model of unilateral cortical critical bone defect of the tibia we used was reported by Melo ^27^ and Ribeiro.^26^ Studies have shown that cortical bone defects in the tibia of SD rats greater than 4 mm × 3 mm cannot be repaired by themselves under normal physiological conditions. We created a bone defect model of 5 mm × 3 mm. The 8‐week‐old SD rats were randomized into 4 groups (n = 12) subjected to treatments (half on week 4 and half on week 12) by Sr‐α‐CSH/n‐HA composite loaded by aspirin of concentrations of 0, 50 μg/ml, 200 μg/ml and 800 μg/ml, respectively. All animals were given a one‐week adaptive feeding before the experiment. In order to avoid the difference in skill between operators, all surgical operations were performed by the same person. Fasting for 4 hours before surgery, intraperitoneal injection of anaesthesia (3% sodium pentobarbital 40 mg/kg) and skin preparation were conducted. Pre‐operative intramuscular injection of penicillin 80 000 units was done to prevent infection. After anaesthesia and skin preparation, the animals were laid on the operating table in side position for routine disinfection, draping and skin cutting. Moulding was performed 0.5 cm below the tibial plateau. The size of the bone defect was 3 mm × 5 mm. During the operation of a grinding brick, the sterile physiological saline was used to rinse its edge, because a high speed brick might result in osteonecrosis because of thermal damage to affect the bone regeneration. Then a 3 mm × 5 mm Sr‐α‐CSH/n‐HA composite previously prepared was filled into the bone defect and rinsed with saline before incision closure. Post‐surgical SD rats were placed on a thermostatic pad until they awoke. The animals were killed at 4 and 12 weeks respectively to harvest the tibias for tissue section staining. All experiments were performed according to the guidelines set by the Institutional Animal Care and Use Committee of Southern Medical University (Guangzhou, China).

### 2.10 Imaging evaluation

Immediately after operation, X‐ray was performed to exam whether the size of a bone defect was appropriate and whether the filling material was loose or not. 3D microarchitecture of the tibia was evaluated using Micro‐CT (µCT 80, Scanco Medical, AG, Switzerland) 4 weeks and 12 weeks post‐surgery. 3D microarchitecture of the tibia bone samples was evaluated. Statistical analysis was performed of bone volume/total volume (BV/TV), trabecular thickness (Tb.Th), trabecular separation (Tb.Sp), trabecular number (Tb.N) in the Micro‐CT region of interest (ROI) in each group of tibial specimens taken at 12 weeks. In order to reduce CT analysis deviation caused by materials and exclude the residual material in defect area, ROI was defined as the regenerative new bone area in the 150 layers (2 mm) above at the edge of the bone defect.

### 2.11 Histological evaluation and quantitative analysis of regenerated bone

The tibial samples were harvested at 4 weeks and 12 weeks post‐surgery, respectively. Bone specimens were fixed in 4% buffered formalin for 24 hours. The specimens were decalcified and embedded in paraffin. Sections of 6 μm thickness were taken from the embedded specimen and stained with HE, Goldner trichrome, immumohistochemical staining (OCN, OST) and TRAP. The numbers of OCN‐positive cells (N.OCN+), OST‐positive (N.OST+) and TRAP‐positive (N.TRAP+) cells were calculated and analysed quantitatively.

### 2.12 Statistical analysis

The results were expressed as mean ± SD of three independent experiments. The significance of variability was analysed by two‐tailed Student's t‐test or one‐way ANOVA followed by Dunnett test. *P* value <0.05 was considered to be significant in all tests. Statistical analyses were performed with SPSS 20 (IBM).

## 3 RESULTS

### 3.1 Characteristics of aspirin‐loaded Sr‐α‐CSH/n‐HA composite

**FIGURE 1:**
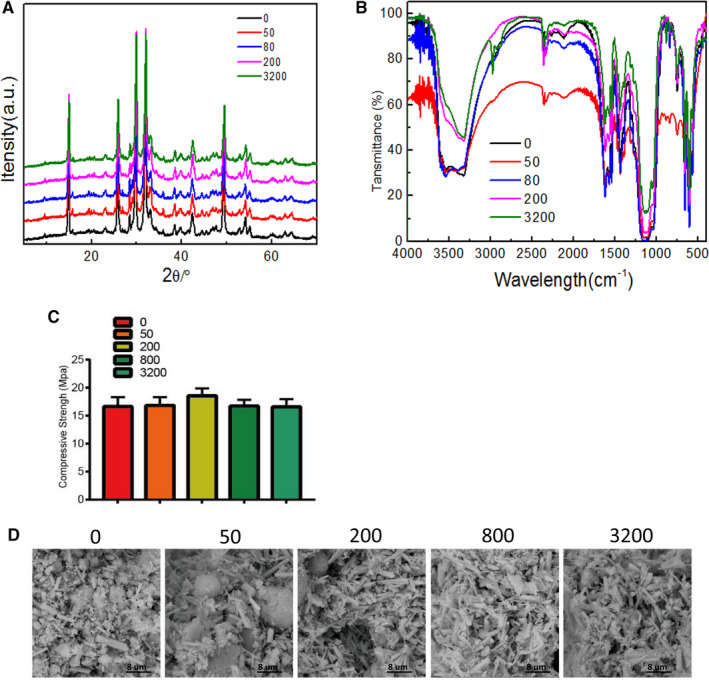
Sr‐α‐CSH/n‐HA composites incorporating 0, 50, 200, 800 and 3,200 ug/mL of aspirin: A, XRD patterns; B, FTIR spectra; C, compressive strength and D, SEM microstructure. Results are expressed as mean ± SD of 5 independent experiments. (*) *P *< 0.05

Results of the X‐ray diffraction (XRD) analysis of the aspirin‐loaded Sr‐α‐CSH/n‐HA composite are shown in Figure 1A. The characteristic crystal diffraction peaks of α‐CSH appeared at 15°, 25°, 30°, 31° and 48°^28^ and the characteristic peak of Strontium was at 24.8°.^29^ n‐HA showed a triplet or broad strong diffraction peak in the range from 31.8° to 34.1°, and an apatite phase diffraction peak at 25.9°.^30^ After aspirin loading, the position of the diffraction peak of the Sr‐α‐CSH/n‐HA composite did not change significantly, but the intensity of the diffraction peak increased with the increased concentration of aspirin loaded, indicating the properties of the Sr‐α‐CSH/n‐HA composite did not change significantly after it was loaded with aspirin. FTIR (Figure 1B) has a high sensitivity for analysis of the inferred material composition, spatial conformation, qualitative and effective functional groups and polymerization crystallization.^31^ As reported, the peak observed in the range from 1622 to 1690 cm^−1^ should correspond to the bending of the O‐H bond, and the peaks in the range from 600 to 660 cm^−1^ and from 1111 to 1140 cm^−1^ correspond to the stretching and bending of the S‐O bond, respectively,^32^ and the peak in the range from 980 to 990 cm^−1^ correspond to (PO3)^2‐^.^33^ After aspirin loading, there was no significant bias in the characteristic diffraction peaks of these bindings, indicating no significant effect on the properties of the Sr‐α‐CSH/n‐HA composite after aspirin loading. The results of the compressive strength test of aspirin‐loaded Sr‐α‐CSH/n‐HA composite are shown in Figure 1C. With the aspirin concentration in the composite material increasing, the compressive strength of the Sr‐α‐CSH/n‐HA composite did not change significantly, and the differences among groups were not statistically significant (*P* > 0.05). SEM (Figure 1D) showed that the aspirin‐loaded Sr‐α‐CSH/n‐HA composite was rough and porous. The crystals were arranged in a short rod shape and a lamellar shape, and no obvious difference was observed among groups. The above results showed that the characteristics of Sr‐α‐CSH/n‐HA composites were not significantly changed after aspirin loading.

### 3.2 Effects of aspirin‐loaded Sr‐α‐CSH/n‐HA composite on proliferation and migration of BMSCs

**FIGURE 2:**
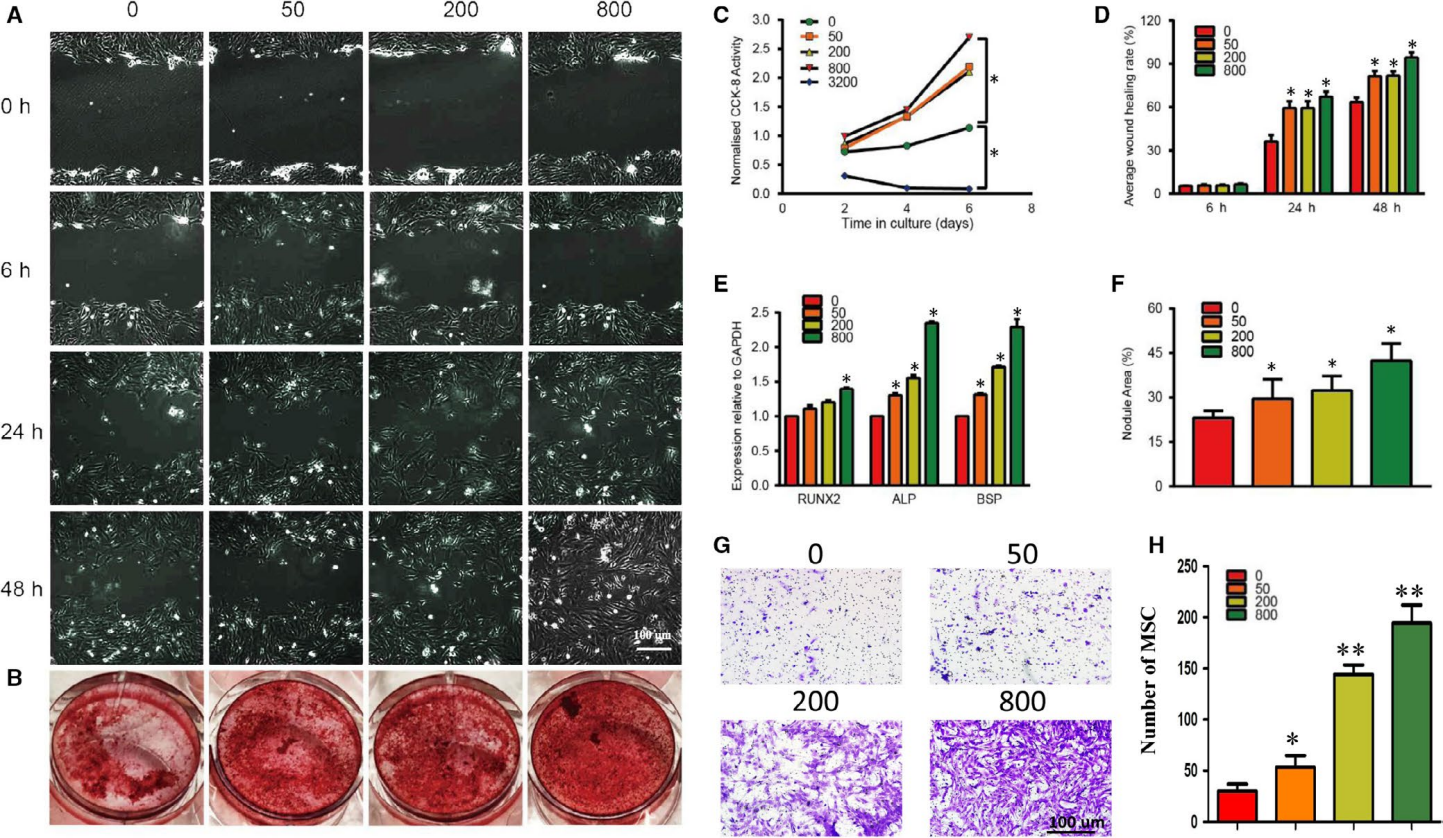
Effects of aspirin‐loaded Sr‐α‐CSH/n‐HA composite extract on proliferation, differentiation and migration of BMSCs in vitro. 0, 50, 200, 800 and 3200 represent the concentrations of aspirin loaded (μg/ml). A, Representative micrographs of BMSCs scratch experiments 0 h, 6 h, 24 h and 48 h after co‐cultivation with aspirin‐loaded Sr‐α‐CSH/n‐HA composite extracts of 0 μg/ml, 50 μg/ml, 200 μg/ml and 800 μg/ml. Scale bar, 100 μm. B, Alizarin red staining. C, Effect of aspirin‐loaded Sr‐α‐CSH/n‐HA composite extracts on cell proliferation (CCK‐8). D, Statistical analysis of scratch healing rates. E, mRNA expression levels of osteogenesis‐related genes (Runx2, ALP and BSP) after BMSCs were treated with aspirin‐loaded Sr‐α‐CSH/n‐HA composite extracts under osteoinductive conditions for two weeks. F, Statistical analysis of mineralized nodules in Alizarin red staining. G‐H, The microscopic observation and quantitative analysis of Transwell assay showed that aspirin‐loaded Sr‐α‐CSH/n‐HA composite extracts induced BMSCs migration. Scale bar, 100 μm. (*P < 0.05. Data are presented as mean ± SD of three independent experiments.）

As shown in Figure 2C, Sr‐α‐CSH/n‐HA loaded with aspirin of concentrations of 50 μg/ml, 200 μg/ml and 800 μg/ml showed a promoting effect on BMSCs proliferation while Sr‐α‐CSH/n‐HA loaded with a high concentration of aspirin (3,200 μg/ml) displayed an inhibitory effect on cell proliferation. Differences between groups were statistically significant (*P* < 0.05). As Sr‐α‐CSH/n‐HA loaded with 3200 μg/ml aspirin led to cytotoxicity for BMSCs, our following experiments did not take this concentration into consideration. The effects of extracts of aspirin‐loaded Sr‐α‐CSH/n‐HA composite on migration of BMSCs were shown in Figure 2A. It was obvious that the BMSCs processed by extracts of aspirin‐loaded Sr‐α‐CSH/n‐HA showed an increasing migration ability than those of Sr‐α‐CSH/n‐HA material in a dose‐dependent manner in the range of this experiment. The statistical analysis of scratch healing rates between groups showed that the differences among groups were statistically significant (Figure 2D) (*P* < 0.05). As the concentration of aspirin increased, the effect of scratch repair increased in the present study. The results of Transwell test were consistent with those of the scratch test. With the increase in aspirin concentration, the migration rate of BMSCs was significantly accelerated (Figure 2G‐H).

### 3.3 Aspirin‐loaded Sr‐α‐CSH/n‐HA stimulates the osteogenesis of BMSCs in vitro

As shown in Figure 2B, when BMSCs were subjected to osteogenic inductive conditions, treatment with aspirin‐loaded Sr‐α‐CSH/n‐HA increased their capability of forming Alizarin red‐positive calcified deposits. Extracts of aspirin‐loaded Sr‐α‐CSH/n‐HA resulted in significantly more Alizarin red‐positive cells compared with those of Sr‐α‐CSH/n‐HA in a dose‐dependent manner (Figure 2F) (*P *< 0.05). As the concentration of aspirin increased, there were more Alizarin red‐positive cells. This was confirmed by up‐regulation of osteogenesis‐related genes (Runx2, ALP and BSP) after BMSCs were treated with extracts of aspirin‐loaded Sr‐α‐CSH/n‐HA in osteoinductive conditions for one week (Figure 2E). Differences among groups were statistically significant (*P* < 0.05). All these data indicated that aspirin‐loaded Sr‐α‐CSH/n‐HA might stimulate the osteogenesis of BMSCs in vitro.

### 3.4 Aspirin‐loaded Sr‐α‐CSH/n‐HA composite promotes critical bone defect regeneration in SD rats

**FIGURE 3:**
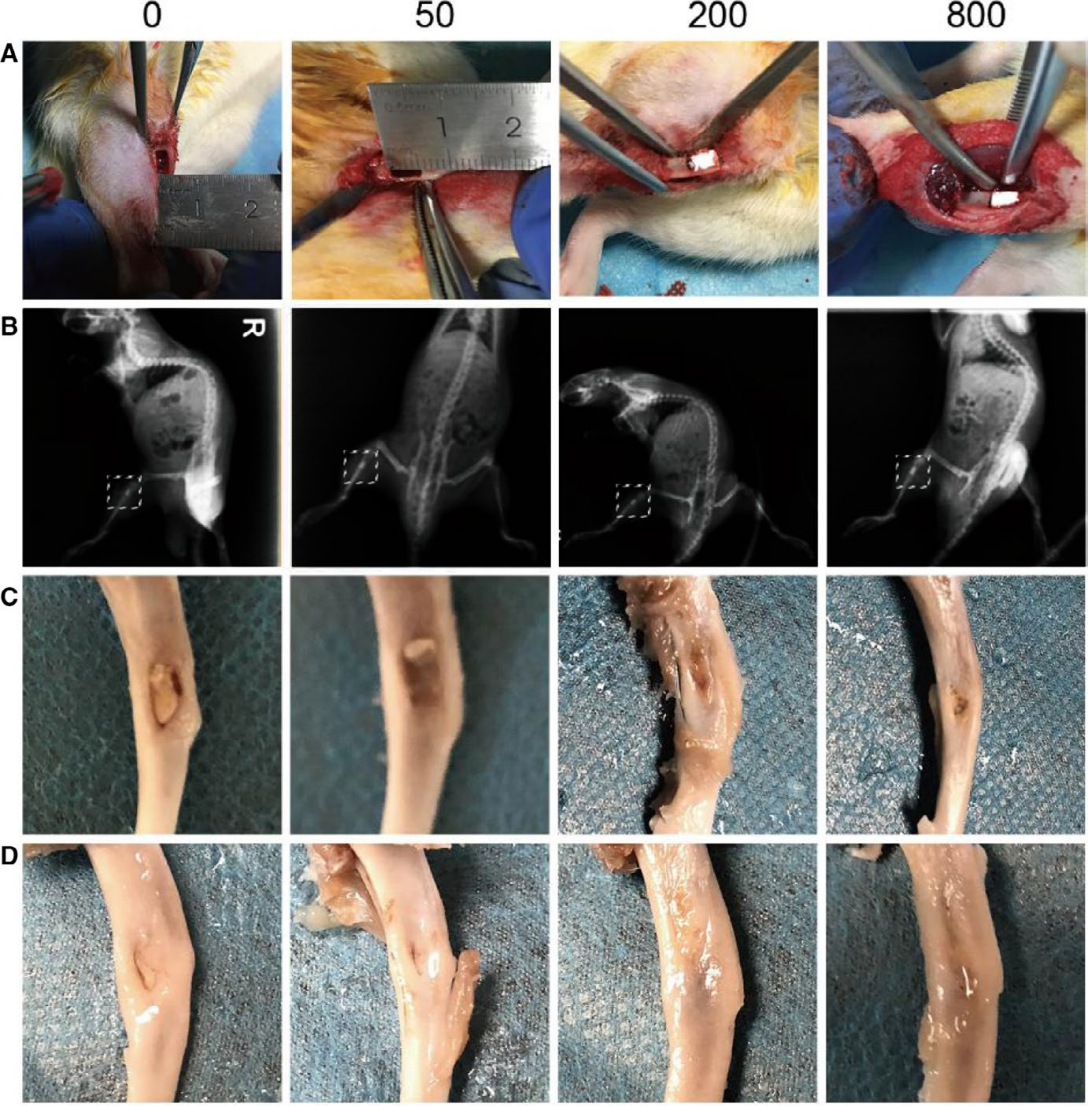
Gross observation. A, Tibia bone defects were freshly created in SD rats. B, X‐ray observation immediately after surgery to detect loosening or shedding of the filling material. C and D, Gross observation of the tibia 4 weeks and 12 weeks after surgery

**FIGURE 4:**
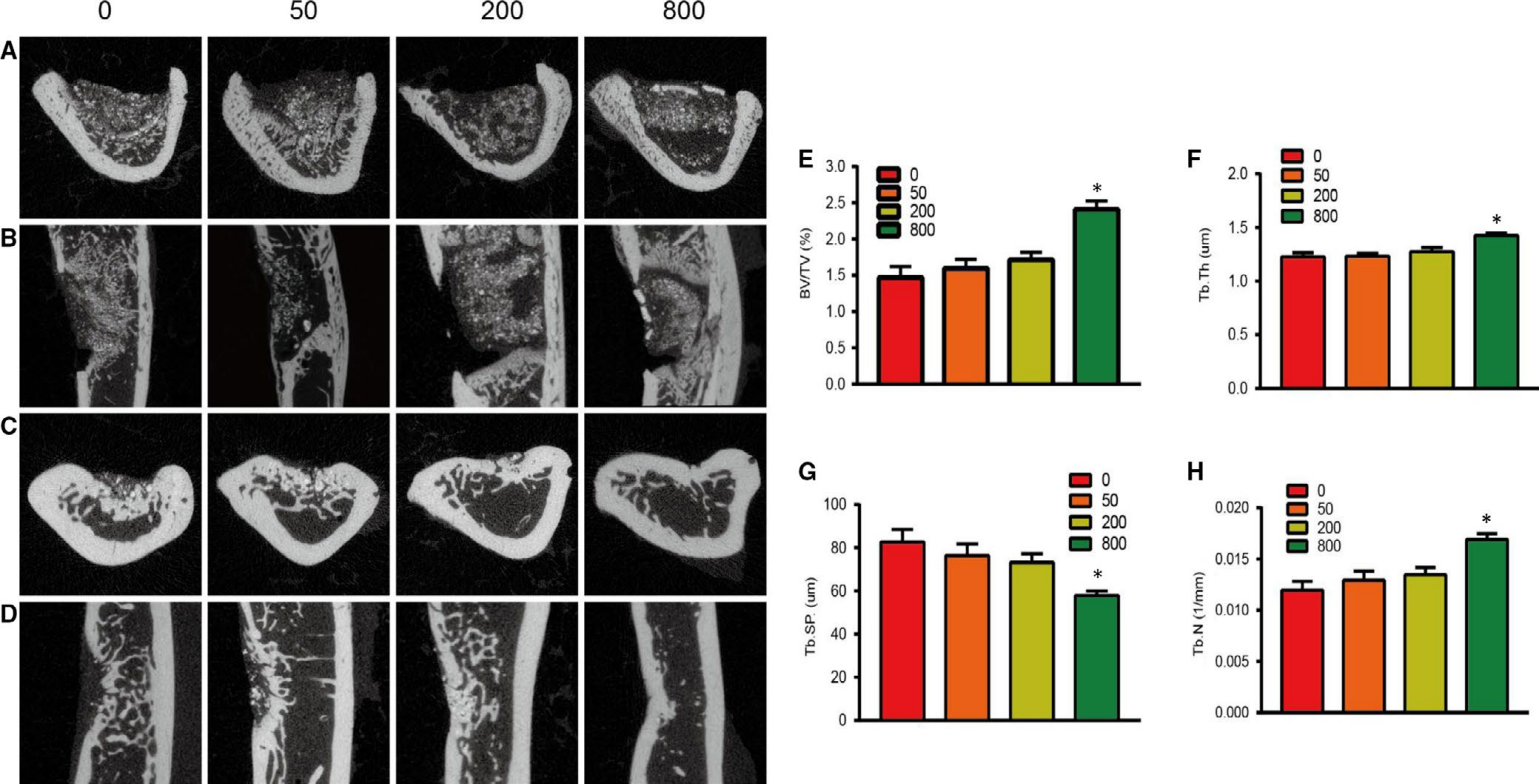
Aspirin‐loaded Sr‐α‐CSH/n‐HA composite promoted defective bone regeneration in SD rats. 0, 50, 200 and 800 represent the concentrations of aspirin loaded (μg/ml). Micro‐CT images of tibia defect areas captured 4 weeks (A and B) and 12 weeks (C and D) post‐surgery. Statistical analysis of BV/TV, Tb.Th, Tb.N and Tb.SP in different groups (E‐H). Results are expressed as mean ± SD and statistical significance is shown as (*) *P* < 0.05

As shown by the X‐ray taken immediately after surgery to observe whether there was loosening or shedding in the aspirin‐loaded Sr‐α‐CSH/n‐HA composite filling the tibial bone defects freshly created in SD rats (Figure 3A and B), the material was well located with no loosening or shedding in all groups. Micro‐CT analyses at 4 weeks (Figure 3C) and 12 weeks (Figure 3D) following surgery of the tibial bone specimens showed good bone quality in all specimens, with no obvious thickening, deformation or obvious signs of infection or pathological fractures. Micro‐CT illustrated that treatment with aspirin‐loaded Sr‐α‐CSH/n‐HA composite significantly increased regeneration of critical bone defects in SD rats. At 4 weeks following surgery, the margin of the defect was still clearly identifiable but there was a certain amount of new bone formation in all the four groups, with the 800 μg/ml group showing the most new bone formation (Figure 4A and B). At 12 weeks following surgery, the margin of the defect was not clearly identifiable due to formation of mineralized tissue (Figure 4C and D). The Sr‐α‐CSH/n‐HA composite group showed the least bone formation while the aspirin‐loaded Sr‐α‐CSH/n‐HA composite groups exhibited formation of a moderate amount of mineralized tissues. The 800 μg/ml group displayed almost full restoration of the defects compared to the Sr‐α‐CSH/n‐HA composite group. Quantitative examination of Micro‐CT images at 12 weeks demonstrated that treatment with aspirin‐loaded Sr‐α‐CSH/n‐HA composite led to significantly higher BV/TV, Tb.Th and Tb.N than treatment with Sr‐α‐CSH/n‐HA material (*P* < 0.05) while treatment with aspirin‐loaded Sr‐α‐CSH/n‐HA led to significantly lower Tb.SP. than treatment with Sr‐α‐CSH/n‐HA material in a dose‐dependent manner (*P* < 0.05) (Figure 4E‐H).

### 3.5 Aspirin‐loaded Sr‐α‐CSH/n‐HA stimulates osteogenesis of BMSCs in vivo

**FIGURE 5:**
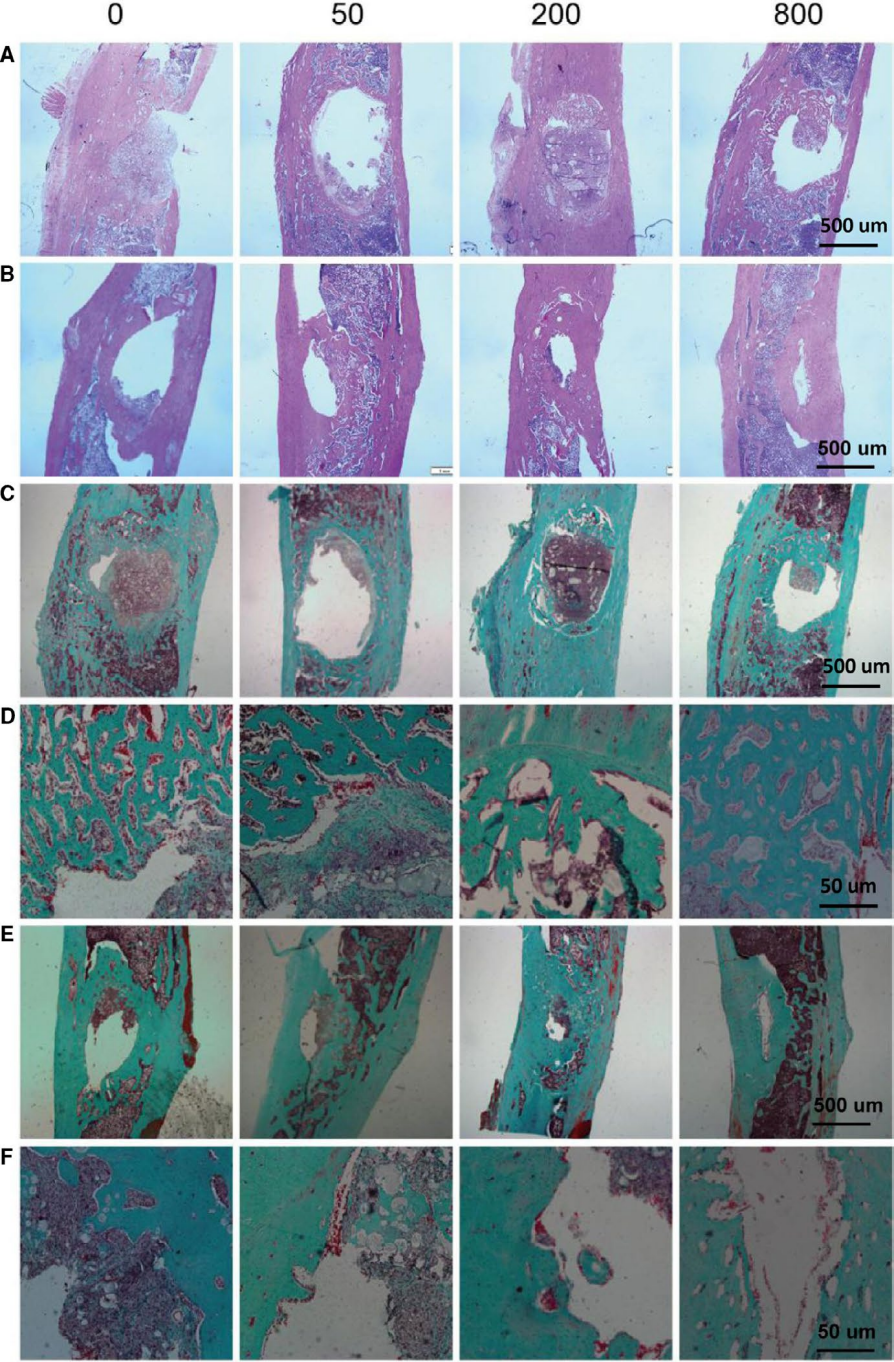
Aspirin‐loaded Sr‐α‐CSH/n‐HA composite promoted tibia bone regeneration in SD rats. Specimens of tibia bone subjected to different treatments were retrieved 4 weeks and 12 weeks post‐surgery. Sections were stained with H&E 4 weeks and 12 weeks post‐surgery, respectively (A‐B: scale bar, 500 μm). Goldner trichrome images 4 weeks post‐surgery (C‐D) and 12 weeks post‐surgery (E‐F); Goldner trichrome images at a lower magnification (C & E: scale bar, 500 μm) and at a higher magnification (D & F: scale bar, 50 μm)

**FIGURE 6:**
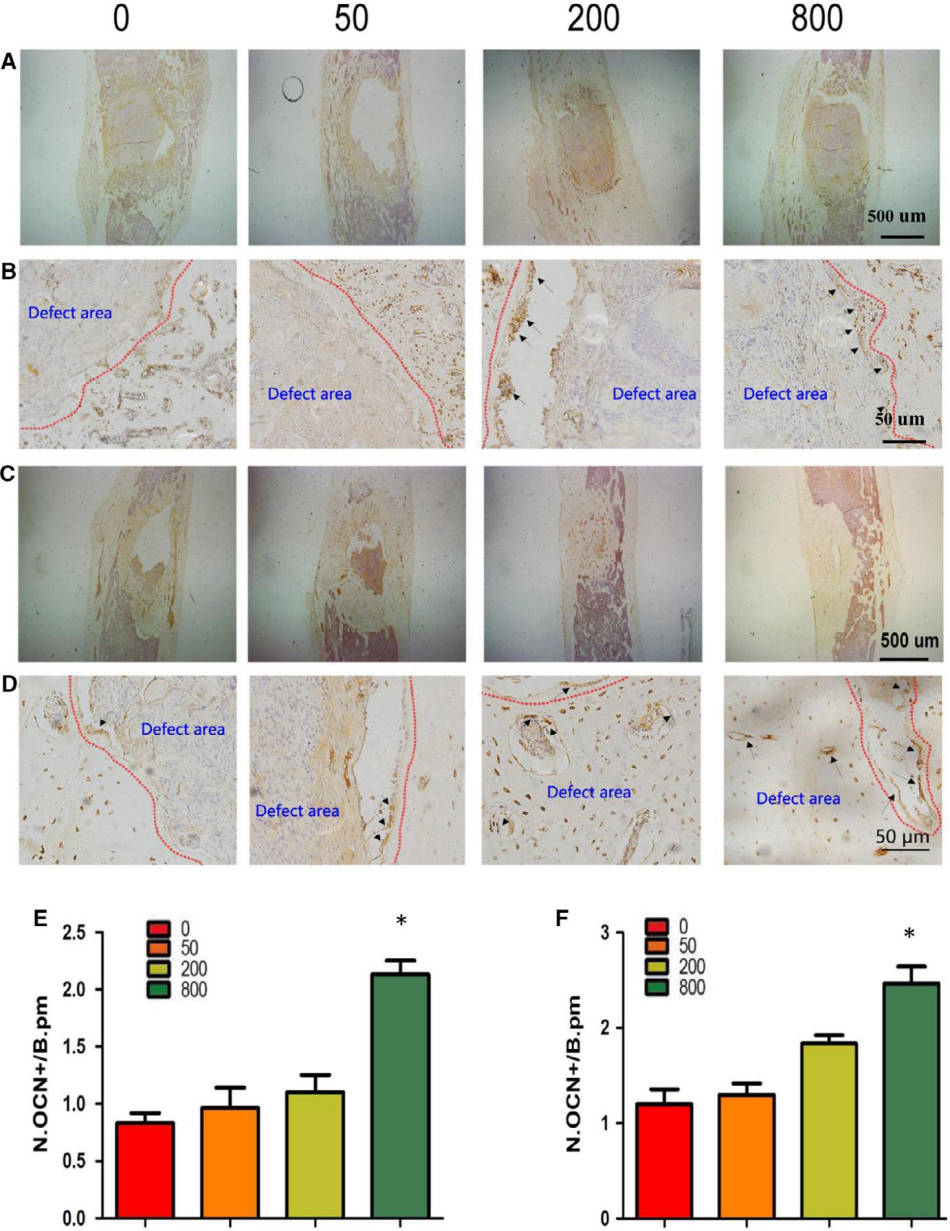
Effects of aspirin‐loaded Sr‐α‐CSH/n‐HA complex on differentiation of BMSCs in vivo. 0, 50, 200 and 800 represent the concentrations of aspirin loaded (μg/ml). Immunohistochemical staining images 4 weeks post‐surgery (A‐B) and 12 weeks post‐surgery (C‐D); Immunohistochemical staining images at a lower magnification (A & C: scale bar, 500 μm) and at a higher magnification (B and D: scale bar, 50 μm). The number of OCN‐positive cells (N.OCN+) on the bone surface was measured as cells per millimetre of perimeter in sections (/B.Pm). Results are expressed as mean ± SD and statistical significance is shown as (*) *P* < 0.05

**FIGURE 7:**
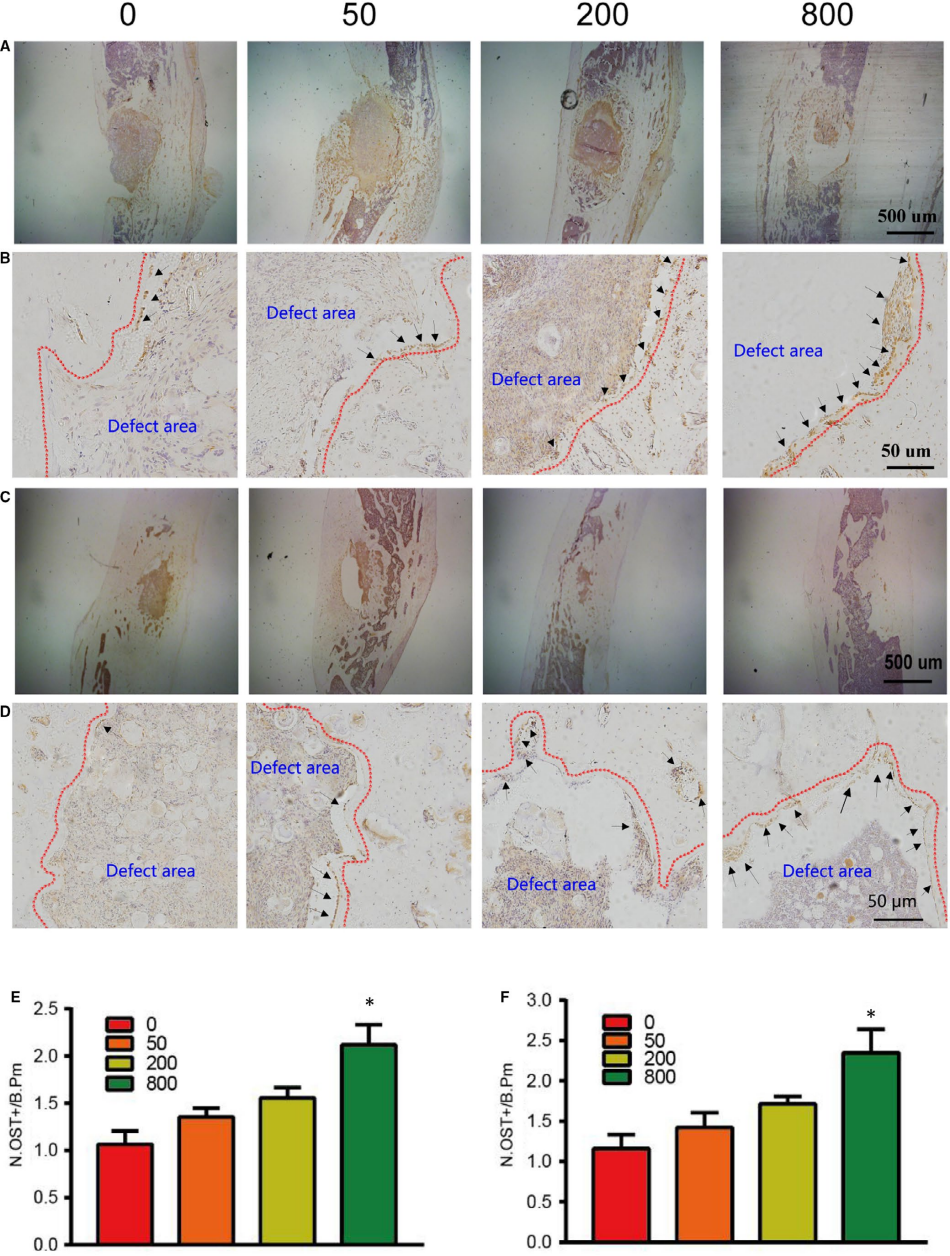
Effects of aspirin‐loaded Sr‐α‐CSH/n‐HA complex on differentiation of BMSCs in vivo. 0, 50, 200 and 800 represent the concentrations of aspirin loaded (μg/ml). Immunohistochemical staining images 4 weeks post‐surgery (A‐B) and 12 weeks post‐surgery (C‐D); Immunohistochemical staining images at a lower magnification (A and C: scale bar, 500 μm) and at a higher magnification (B and D: scale bar, 50 μm). The number of Osterix‐positive cells (N.OST+) on the bone surface was measured as cells per millimetre of perimeter in sections (/B.Pm). Results are expressed as mean ± SD and statistical significance is shown as (*) *P* < 0.05

**FIGURE 8:**
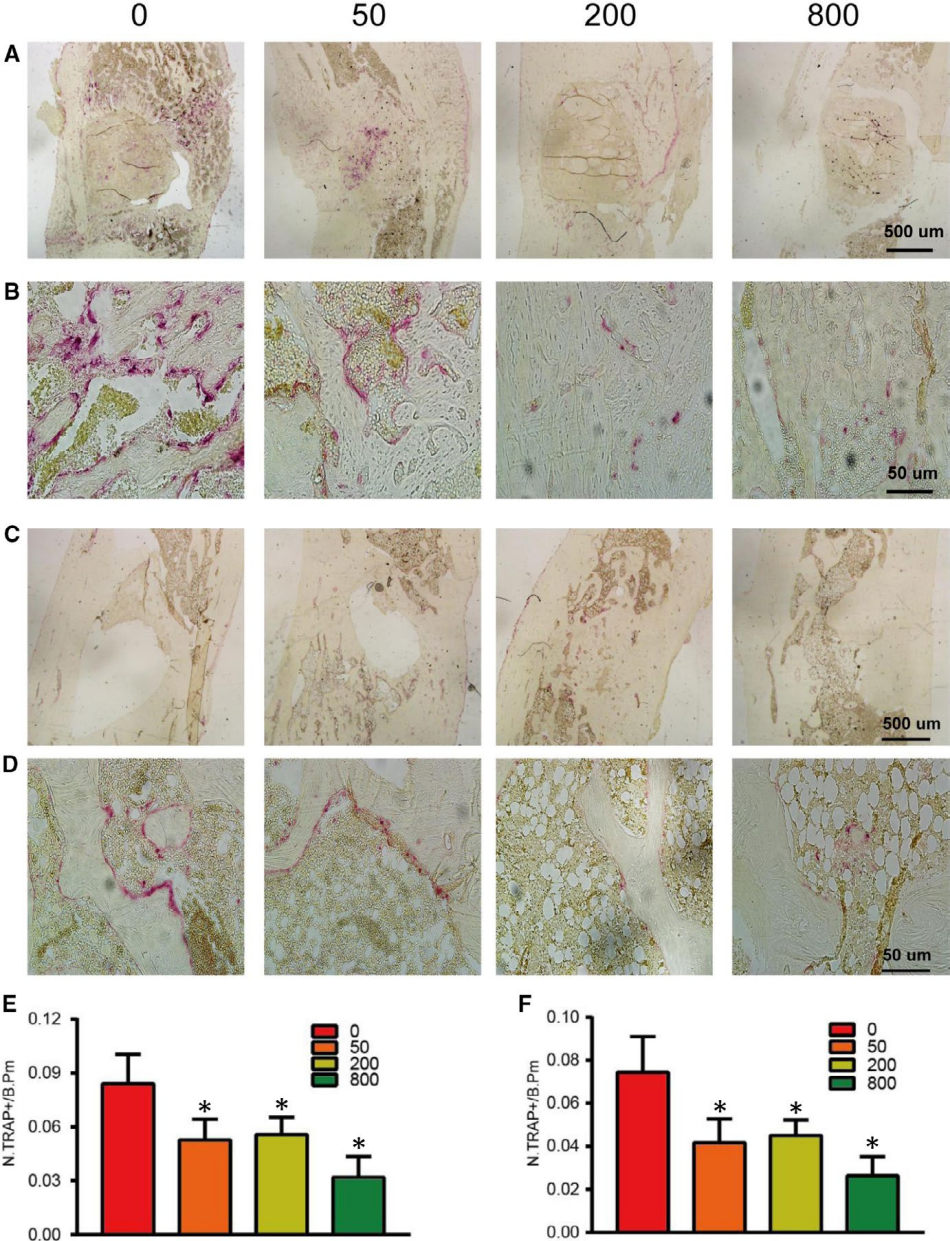
Effects of aspirin‐loaded Sr‐α‐CSH/n‐HA complex on the activity of osteoclast in vivo. 0, 50, 200 and 800 represent the concentrations of aspirin loaded (μg/ml). Trap staining images 4 weeks post‐surgery (A‐B) and 12 weeks post‐surgery (C‐D); Immunohistochemical staining images at a lower magnification (A & C: scale bar, 500 μm) and at a higher magnification (B & D: scale bar, 50 μm). The number of Trap‐positive cells (N.TRAP+) on the bone surface was measured as cells per millimetre of perimeter in sections (/B.Pm). Results are expressed as mean ± SD and statistical significance is shown as (*) *P* < 0.05

At 4 weeks and 12 weeks, sections of tibial specimens were harvested for histological examination using HE staining (Figure 5A and B), Goldner trichrome (Figure 5C‐F), immunohistochemical staining (OCN, OST) (Figures 6 and 7) and TRAP staining (Figure 8). Images at lower magnifications showing the whole defect area (Figure 5A) demonstrated that Sr‐α‐CSH/n‐HA composite group had the least bone formation, aspirin‐loaded Sr‐α‐CSH/n‐HA composite group had formation of a moderate amount of mineralized tissues, and the 800 μg/ml Sr‐α‐CSH/n‐HA group displayed almost full restoration of the defects. Images at a higher magnification (Figure 5B) demonstrated more detailed information about the formation of mineralized tissues. Furthermore, Goldner trichrome (Figure 5C‐F) indicated that 800 μg/ml Sr‐α‐CSH/n‐HA group had more new bone formation compared to the Sr‐α‐CSH/n‐HA composite group. This was also confirmed by immunohistochemical staining (OCN, OST) (Figures 6 and 7) and TRAP staining (Figure 8). The 800 μg/ml Sr‐α‐CSH/n‐HA group had more OCN, OST‐positive cells and fewer TRAP‐positive cells compared with Sr‐α‐CSH/n‐HA composite group.

## 4 DISCUSSION

It is a great challenge to develop a promising BGS which can promote high quality new bone formation.^20^ The present study demonstrated that the aspirin‐loaded Sr‐α‐CSH/n‐HA composite was capable of promoting regeneration of critical bone defects in SD rats because topical administration of aspirin held a great advantage of promoting osteogenesis of BMSCs. The aspirin‐loaded Sr‐α‐CSH/n‐HA may also have some therapeutic effects on bone defects caused by tumour resection, malformation, sports injury and infection.^1^, ^2^, ^3^, ^4^

The present study started with analyses of the crystal structure and physical characteristics of aspirin‐loaded Sr‐α‐CSH/n‐HA composite using XRD, FTIR, SEM and CST. The principle of XRD technology is to understand the internal structure of the materials to be examined by analysing the broad spectrum of specific materials. Through statistical analysis of XRD results, qualitative and quantitative analysis of materials can be performed in a certain sense.^34^ FTIR has a high sensitivity in analysis of material composition, spatial conformation, qualitative and effective functional groups and polymerization crystallization.^33^ SEM is used to observe microstructure of the material, such as roughness and porous arrangement.

Structural support is an important property of BGS. The compressive strength of the BGS is very important in supporting the defective part during loading. An insufficient compressive strength will lead to breakage of an implant or outer frame, causing a failure in repair. The compressive strength of all our samples of aspirin‐loaded Sr‐α‐CSH/n‐HA composite exceeded that of normal cancellous bone (generally between 2‐10 MPa), indicating that our new material can fully meet the clinical requirement for strength. We further found no significant changes in the crystal structure and physical characteristics between Sr‐α‐CSH/n‐HA composite and aspirin‐loaded Sr‐α‐CSH/n‐HA composite, indicating aspirin‐loaded Sr‐α‐CSH/n‐HA is a qualified BGS in compressive strength.

Inclusion of aspirin in bone replacement materials has been studied by many scholars. Du et al ^35^ found that platelet‐rich fibrin/aspirin complex provided a suitable physiological microenvironment to enhance adhesion and viability of mesenchymal stem cells and protected mesenchymal stem cells from the host immune system by anti‐inflammatory effects in an early stage of transplantation. Zhang et al ^36^ who studied the repair of skull defects in rats by aspirin‐loaded collagen‐chitosan membranes found that aspirin‐loaded chitosan nanoparticles had a better effect compared to controls. Fang et al ^37^ showed that aspirin‐loaded hydrogels promoted bone regeneration better. All these have proved that aspirin can be used as a loaded drug in bone transplantation to safely and effectively enhance the effects of BGS on bone regeneration and repair.

Similarly, our study demonstrated that Sr‐α‐CSH/n‐HA composite loaded with aspirin of a safe range of concentrations was capable of promoting osteogenesis both in vitro and in vivo with no cytotoxicity to BMSCs and had a better effect on regeneration of critical bone defects than Sr‐α‐CSH/n‐HA material in SD rats. What is more, Sr‐α‐CSH/n‐HA loaded with 800 μg/ml aspirin had the best effect on osteogenesis of BMSCs, though a previous research showed that the concentrations of aspirin ranging from 50 μg/ml to 200 μg/ml had the strongest effect on osteogenic differentiation of BMSCs and that the concentration of aspirin over 400 μg/ml might have a certain toxic effect on BMSCs.^23^ Their finding seems contradictory to ours, but there might have been something uncertain in it. Although we used 800 μg/ml aspirin solution to dissolve the material, in preparation of composite or extract of aspirin‐loaded Sr‐α‐CSH/n‐HA, the concentration of aspirin might have been diluted to some extent. Further experimental verification is required to determine the actual aspirin concentration in the extract.

Some studies reported different effects of aspirin on bone formation. Lin et al^38^ found that aspirin treatment significantly prevented bone loss by increasing bone formation, but did not show any significant improvement in bone mechanical property. William et al^39^ showed that aspirin had a similar dose‐dependent effect on bone healing in a typical dose range. The increasing concentration of aspirin significantly affected fracture healing, especially when the dose was greater than that (325 mg) for a human body.

It has been accepted that host systemic conditions account for, at least in part, the imbalance in bone remodelling in the process of implant‐associated infection (IAI) though the bacterial infection is thought to be one of the dominant factors. The current therapeutic strategy for the management of IAI, based on documented scientific literature, is still a major clinical problem. Clinical treatment of implant‐related infections in orthopaedics is very difficult and requires continuous debridement. Thorough debridement will inevitably lead to certain bone defects. As our previous studies have shown that aspirin can alleviate IAI and increase bone formation in infected bone, we believe that mechanical debridement in conjunction with aspirin‐loaded Sr‐α‐CSH/n‐HA might have favourable therapeutic effects on bone defects associated with infection. Considering the fact that aspirin has been used as a NSAID for decades with known side effects, local administration of aspirin should possess fewer side effects than other strategies, such as use of genetically modified stem cells or systemic infusion of regulatory T cells. Future studies should investigate the potentials of aspirin‐loaded Sr‐α‐CSH/n‐HA in treatment of critical bone defects caused by IAI.

Our research has certain limitations. First, we did not detect the actual concentration of aspirin in the extract. We believed that testing the aspirin concentration alone was not reasonable enough to explain the effect of composite material in the treatment of bone defect because the synergy effect on osteogenesis should be associated with Sr‐α‐CSH/n‐HA composite and aspirin. However, as the safety of biomaterials must be our priority, we first excluded the group showing a cytotoxic effect by aspirin‐loaded Sr‐α‐CSH/n‐HA composite. As our animal experiments showed that 800 μg/ml had the best effect on bone defect repair and 3200 μg/ml aspirin in the cell experiment inhibited the proliferation of BMSCs, we just inferred that the actual concentration of aspirin should have been greater than 200 μg/ml according to previous reports. Another shortcoming of ours is that we only studied the biological and osteogenic properties of aspirin‐loaded Sr‐α‐CSH/n‐HA composite but did not further investigate their specific mechanisms and signalling pathways. Further experiments are required.

## 5 CONCLUSIONS

Aspirin‐loaded Sr‐α‐CSH/n‐HA may be capable of promoting tibia bone regeneration in SD rats. Local administration of aspirin, coupled with Sr‐α‐CSH/n‐HA, has a twofold effect on regeneration of critical bone defects, alleviating inflammatory response at sites of disease, increasing bone formation and, to some extent, preventing IAI.

## CONFLICT OF INTEREST

The authors confirm that there are no conflicts of interest.

## AUTHORS’ CONTRIBUTIONS

Hanjun Qin: Formal analysis (equal); Methodology (equal); Software (equal); Writing‐original draft (equal); Writing‐review & editing (equal). Yi Jiang: Formal analysis (equal); Investigation (equal); Methodology (equal); Software (equal); Writing‐original draft (equal). Haoyang Wan: Formal analysis (supporting); Methodology (supporting); Software (supporting); Writing‐review & editing (supporting). Jun Yang: Formal analysis (supporting); Investigation (supporting); Methodology (supporting); Writing‐review & editing (supporting). Qi Yu: Investigation (supporting); Methodology (supporting); Resources (supporting); Software (equal). Mo Jiang: Data curation (equal); Project administration (equal); Supervision (equal). Bin Yu: Conceptualization (equal); Data curation (equal); Funding acquisition (lead); Investigation (equal); Methodology (equal); Project administration (equal); Supervision (lead); Validation (lead); Visualization (equal); Writing‐original draft (supporting); Writing‐review & editing (equal).
